# Les lentigines

**DOI:** 10.11604/pamj.2019.33.190.15989

**Published:** 2019-07-12

**Authors:** Fatima-Zahra Agharbi

**Affiliations:** 1 Centre, Hôpital Civil Tétouan, Tétouan, Maroc

**Keywords:** Lentigines, neurofibromatose, hypetrmélanocytose, Lentigines, neurofibromatosis, hypermelanocytosis

## Abstract

Chancroid (also known as soft chancre and ulcus molle) is a sexually transmitted disease (STD) due to the Ducrey’s bacillus (or Haemophilus ducreyi) characterized by chancre at the site of ulcerated inoculation associated with lymphadenopathy. The disease manifests as a small pinkish papule at the site of penetration of the bacterium. After an incubation period ranging from 24 hours to 15 days (on average 5 days). The lesion rapidly evolves into a more or less extended pinkish, painful, deep ulcer with very inflamed and sharp edges and a ragged appearance. The lymphadenopathies usually occur 2-3 weeks after the contact. They are often unilateral and can evolve into ulcers with pus discharge at the level of the skin. Some complications can occur: penile gangrene, extended gangrene of the skin, local superinfection, association with other sexually transmitted diseases. Bacterium can be identified by microscopic examination of a smear of the chancre-like ulcer, more rarely by fine-needle puncture biopsy of a lymphadenopathy. Giemsa or Pappenheim coloration allows identification of the germ. Treatment is based on azithromycin (1g per os in a single dose) or ceftriaxone (250mg administered intramuscularly in a single dose). We report the case of a 30-year old man with well-defined deep scrotum ulcer with necrotic center which occurred 1 week after unprotected sexual intercourse. Haemophilus ducrey has been detected by culture and the patient underwent Azithromycin therapy with good outcome.

## Image en médecine

Lentigines sont des macules hyperpigmentées, de petite taille (1 à 3mm), qui se distinguent des éphélides: par leur teinte plus foncée (brune ou noire) et par leur absence de modification après exposition solaire. Généralement régionales, elles peuvent se répartir sur toute la surface du tégument et/ou sur les muqueuses. Elles peuvent apparaître dans l'enfance ou plus tardivement au cours de la vie. Ces lentigines apparaissent parfois très rapidement sur un mode éruptif. Les lentigines correspondent à une hypermélanocytose épidermique, avec présence occasionnelle de grains de mélanine géants, dénommés macromélanosomes. Les lentigines sont souvent isolées. Parfois, elles s'intègrent dans des syndromes complexes, à expression multiviscérale, et représentent de ce fait des marqueurs ectodermiques utiles, permettant d'évoquer le diagnostic de ces affections. Ces différentes affections sont la lentiginose centrofaciale neurodysraphique de Touraine, la lentiginose périorificielle de Peutz-Jeghers-Touraine, la lentiginose multiple (syndrome LEOPARD), le Complexe de Carney et le Xeroderma pigmentosum. Nous rapportons l'observation d'un jeune de 30 ans sans antécédents pathologiques notables qui présente des lentigines axillaires. L'examen clinique trouvait des neurofibromes sous cutanés et cutanés et de multiples tâches café au lait ce qui a permis de retenir le diagnostic de neurofibromatose.

**Figure 1:**
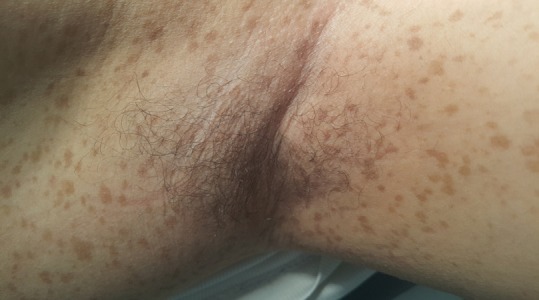
Lentigines axillaires

